# Imaging of shoulder arthroplasties and their complications: a pictorial review

**DOI:** 10.1186/s13244-019-0788-5

**Published:** 2019-10-08

**Authors:** Damien Combes, Romain Lancigu, Patrick Desbordes de Cepoy, Filippo Caporilli-Razza, Laurent Hubert, Louis Rony, Christophe Aubé

**Affiliations:** 1 Radiology Department, Angers University Hospital, 4 rue Larrey, 49933 Angers, France; 2 Orthopedic and Trauma Unit, Angers University Hospital, 4 rue Larrey, 49933 Angers, France

**Keywords:** Shoulder, Arthroplasty, Complications, Imaging

## Abstract

Currently, an increasing number of patients benefit from shoulder prosthesis implantation. Radiologists are therefore more often confronted with imaging examinations involving shoulder arthroplasty, whether during a dedicated examination or incidentally. Standard radiography is the first-line imaging modality in the follow-up of these implants, before the possible use of cross-sectional imaging modalities (computed tomography and magnetic resonance imaging), ultrasound, or nuclear medicine examinations. Shoulder arthroplasties are divided into three categories: reverse shoulder arthroplasty, total shoulder arthroplasty, and partial shoulder joint replacement (including humeral hemiarthroplasty and humeral head resurfacing arthroplasty). Each of these prostheses can present complications, either shared by all types of arthroplasty or specific to each. Infection, periprosthetic fractures, humeral component loosening, heterotopic ossification, implant failure, and nerve injury can affect all types of prostheses. Instability, scapular notching, and acromial fractures can be identified after reverse shoulder arthroplasty implantation. Glenoid component loosening and rotator cuff tear are specific complications of total shoulder arthroplasty. Progressive wear of the native glenoid is the only specific complication observed in partial shoulder joint replacement. Knowledge of different types of shoulder prostheses and their complications’ radiological signs is crucial for the radiologist to initiate prompt and adequate management.

## Key points

- Three main types of shoulder arthroplasty may be encountered, each corresponding to specific clinical situations: reverse shoulder arthroplasty, total shoulder arthroplasty, and partial shoulder joint replacement.
- Shared complications by all types of shoulder prosthesis are fortunately rare but must imperatively be recognized early to initiate prompt and specific treatment.
- Glenoid component loosening is the most frequent complication of total shoulder arthroplasty.
- The most common complication after reverse shoulder arthroplasty is instability, ordinarily in an anterosuperior direction.
- Progressive wear of the native glenoid is the sole complication of partial shoulder joint replacement.

## Introduction

In recent years, an increasing number of patients around the world benefit from the shoulder prostheses implantation. This is allowed by progressing functional outcomes, as well as gradual extensions of shoulder arthroplasty indications.

These indications concern on one hand progressively younger patients (especially in traumatology, e.g., proximal humeral fractures with impossibility of stable osteosynthesis), and on the other hand an increasing number of elderly subjects remaining autonomous and thus eager to maintain satisfactory articular mobility and function. Since the first shoulder prosthesis implantation by Péan in 1893 for tuberculous arthritis [1, 2], development of Neer arthroplasties in the 1950s-1970s [3], and development of Grammont reverse shoulder arthroplasty in the 1980s-1990s [4], shoulder joint implants and their imaging exploration have made considerable progress. Therefore, radiologists will more frequently encounter shoulder arthroplasty imaging, either during a dedicated examination or incidentally. The aim of this article is to offer an iconographic review of normal and pathological aspects of different shoulder prostheses, to familiarize and sensitize the radiologist to the various complications of these arthroplasties, whether shared or specific, that they may encounter in daily practice.

## Normal aspects of different shoulder prostheses

### Reverse shoulder arthroplasty

Reverse shoulder arthroplasty (RSA) is a semi-constrained type of prosthesis with depression and medialization of the glenohumeral rotation center. This allows restoration of deltoid tension and thus abduction and elevation of the arm by the isolated deltoid contraction, without rotator cuff action. Proper deltoid function is therefore required for RSA. The principal indication of this type of implant is rotator cuff arthropathy. Recently, RSA has been developed in trauma surgery for three- to four-part proximal humerus fractures in elderly or osteoporotic patients [5].

**Fig. 1:**
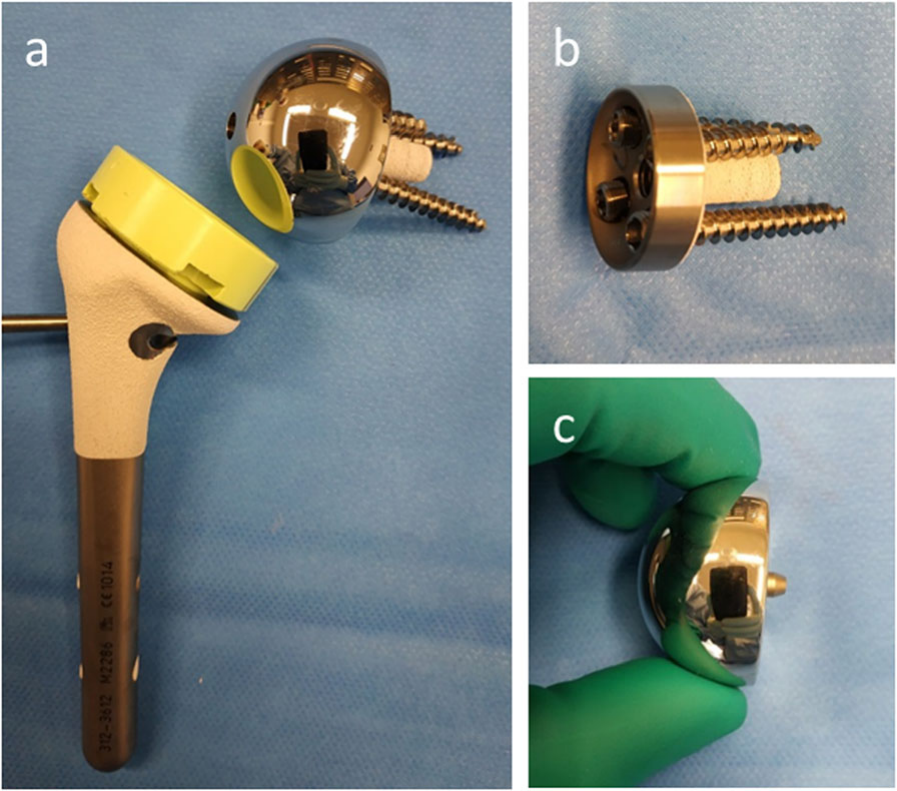
Photograph of a reverse shoulder arthroplasty before implantation in the operating room. **a** Complete prosthesis. **b** Metaglene. **c** Glenosphere

RSA usually consists of four implants (Fig. 1): a humeral component, a polyethylene insert, the glenosphere, and the metaglene. The humeral component is formed by a stem (monoblock or modular) either cemented or uncemented and a cup-shaped proximal portion on which is lined with a polyethylene cup-shaped insert as well. The glenoid components are modular and consist of a base (metaglene) on which is fixed a hemisphere (glenosphere). The metaglene is uncemented and is secured by locking and non-locking screws to the native glenoid. The glenosphere is secured to the base plate of the metaglene by a central screw. This base plate usually has a roughened and coated internal surface to facilitate fixation. Thus, RSA reverses the ball and socket of the shoulder joint [6].

**Fig. 2:**
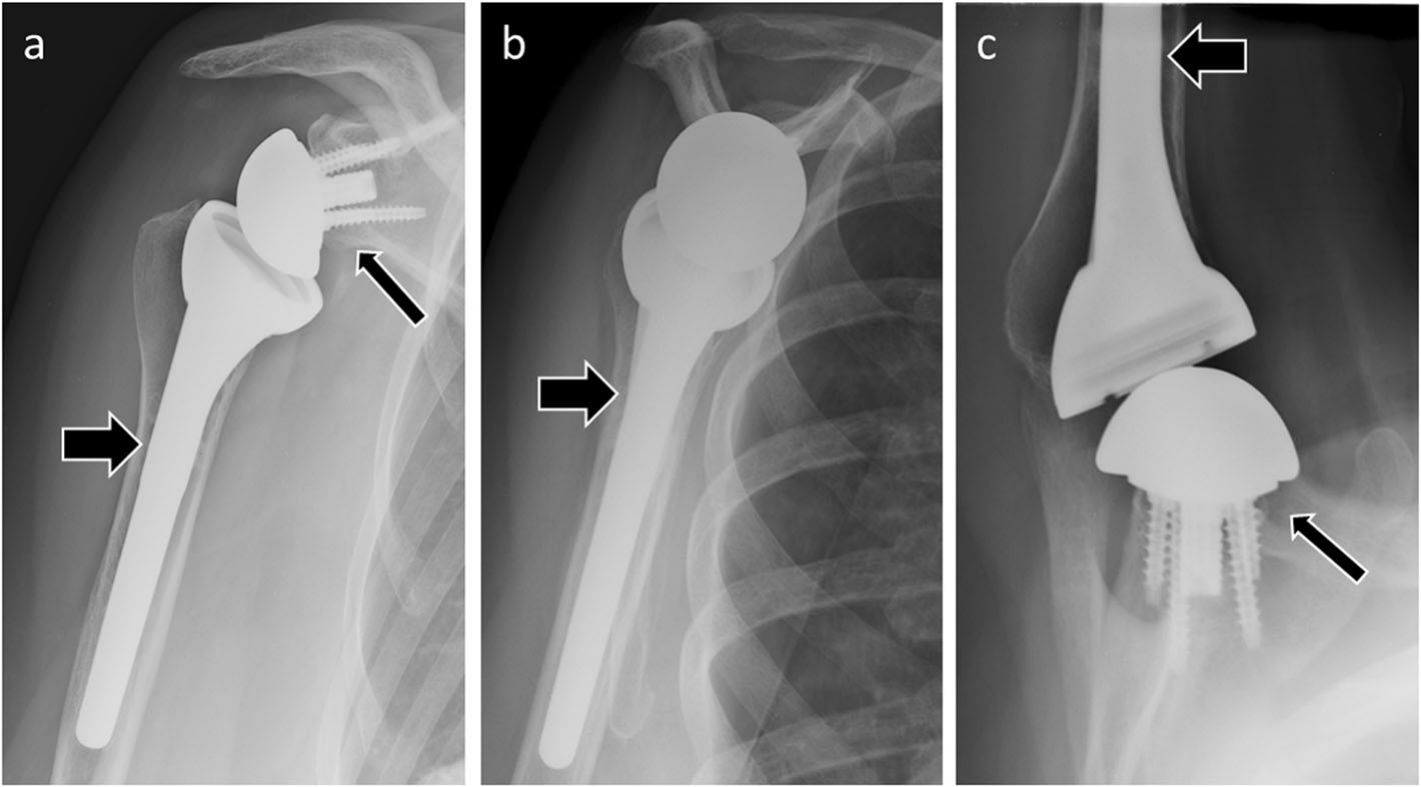
Anteroposterior (**a**), scapular Y (**b**), and axillary (**c**) radiographs of normal findings in a patient with reverse shoulder arthroplasty. The humeral prosthetic stem (thick arrows) is centered within the humeral shaft. The glenosphere (thin arrows) is placed flush with the native glenoid (or slightly lowered) and the humeral component is centered on the glenosphere

At postoperative imaging, the glenosphere should be placed flush with the native glenoid (or even in a lowered position), and the humeral component should be centered on the glenosphere proximally and within the humeral shaft distally (Fig. 2). The amount of space between the glenosphere and the proximal portion of the humeral component is variable, because the radiolucent polyethylene inserts have a relatively wide range of thicknesses and design allowing more or less constraint of the articulation and optimizing stability [7].

### Total shoulder arthroplasty

The aim of total shoulder arthroplasty (TSA) is to replace the glenohumeral joint in a close to native anatomical situation, interesting both the upper end of the humerus and the glenoid. This technique necessitates sufficient glenoid bone stock to ensure glenoid implant fixation. Its main indications are osteoarthritis, aseptic destructive arthropathy (rheumatic, metabolic), and humeral head avascular necrosis with glenoid alteration. TSA can also be performed in the revision of partial joint replacement failure. Humeral head ascension associated with massive rotator cuff tear is a contraindication to TSA; indeed, anatomical joint replacement is based primarily on native soft tissue structures for mobility and longevity. Therefore, it is necessary to ensure beforehand rotator cuff integrity and absence of glenoid wear [5].

TSA includes a humeral component and a glenoid component. The humeral component can be stemmed, stemless (mid-head resection with a metaphyseal anchored component), or resurfacing (surface replacement). There are two main types of glenoid component: cemented polyethylene and metal-backed (less used). They usually have a keel or pegs for fixation. The radiolucent polyethylene glenoid component contains a radiopaque marker within its central peg allowing postoperative radiographic identification. Both glenoid and humeral components can be cemented in place or uncemented [6].

**Fig. 3:**
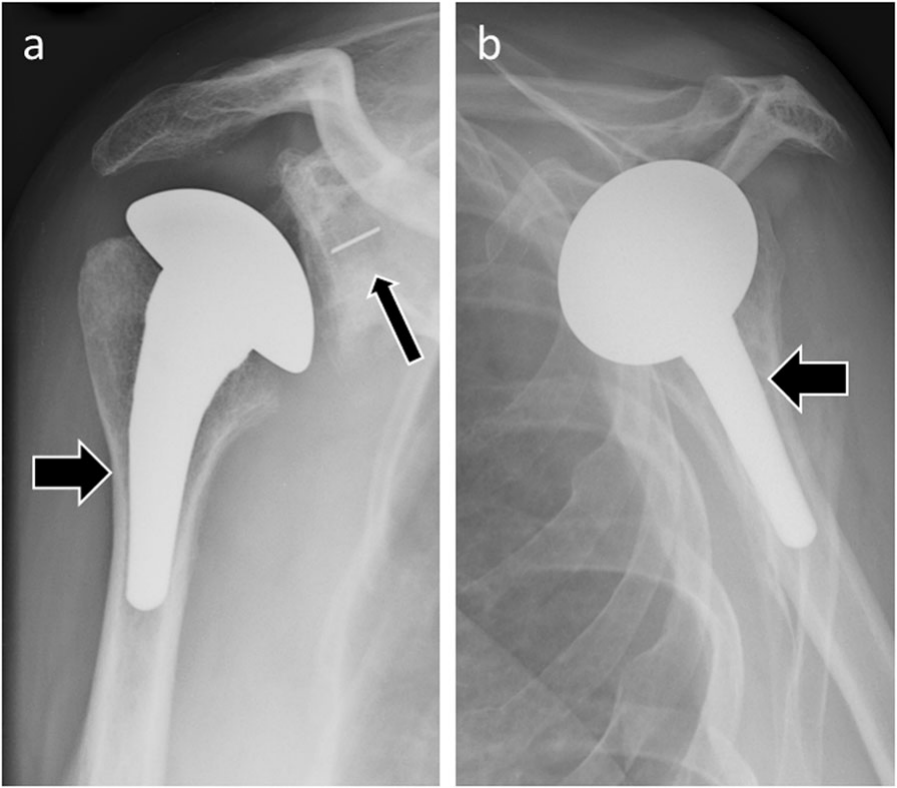
Anteroposterior (**a**) and scapular Y (**b**) radiographs of normal findings in a patient with total shoulder arthroplasty. The humeral prosthetic stem (thick arrows) is centered within the humeral shaft. The humeral prosthetic head is centered within the glenoid implant (thin arrow), with a smooth arc between the cortex of the medial humeral calcar and that of the inferior glenoid and scapular neck

**Fig. 4:**
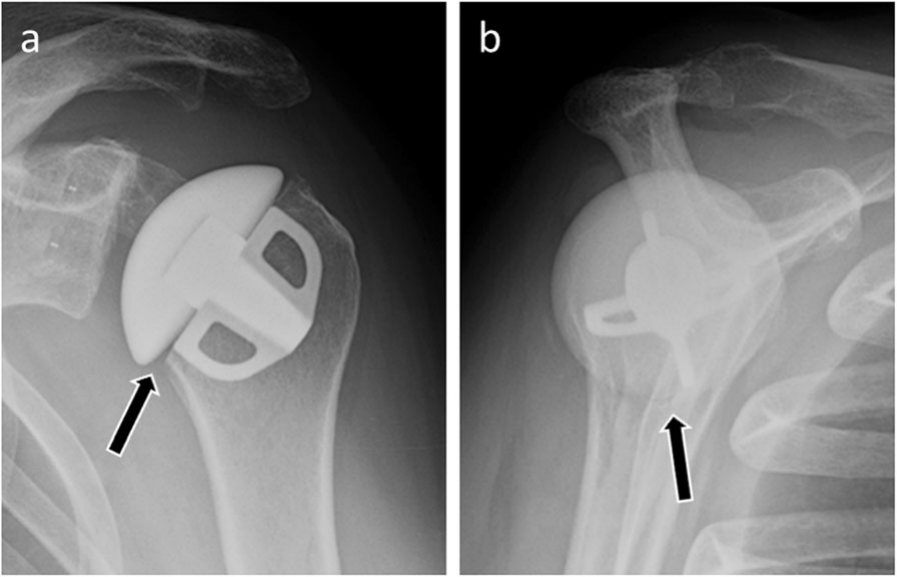
Anteroposterior (**a**) and scapular Y (**b**) radiographs of normal findings in a patient with total shoulder arthroplasty. Note the stemless humeral component (arrows)

On normal postoperative radiographs, the glenoid component should have 0° version and inclination. The humeral head component is centered within the glenoid component, with a smooth arc between the cortex of the medial humeral calcar and that of the inferior glenoid and scapular neck. The humeral prosthetic stem should be centered within the humeral shaft [8] (Figs. 3 and 4).

### Partial shoulder joint replacement

Partial shoulder joint replacement consists of a humeral hemiarthroplasty or a humeral head resurfacing arthroplasty. This type of shoulder prosthesis is used when the glenoid is intact or scantily worn, or conversely when the wear is excessive, making glenoid implantation impossible. Humeral hemiarthroplasty is mainly indicated in avascular necrosis of the humeral head without secondary osteoarthritis, post-traumatic humeral head deformation, rheumatoid arthritis, and joint fracture at high risk of osteonecrosis and with impossibility of stable osteosynthesis but sufficient rotator cuff trophicity. In proximal humerus fractures, surgical fixation of tuberosity must be excellent in order to obtain a functional rotator cuff. When this is compromised, surgeons are more inclined to consider RSA, since it does not require an intact rotator cuff, especially in elderly and osteoporotic patients. Indications of humeral head resurfacing arthroplasty are similar to hemiarthroplasty, but these implants are preferentially used in young patients with more contained humeral head abnormalities and sufficient metaphyseal humeral bone stock [5].

**Fig. 5:**
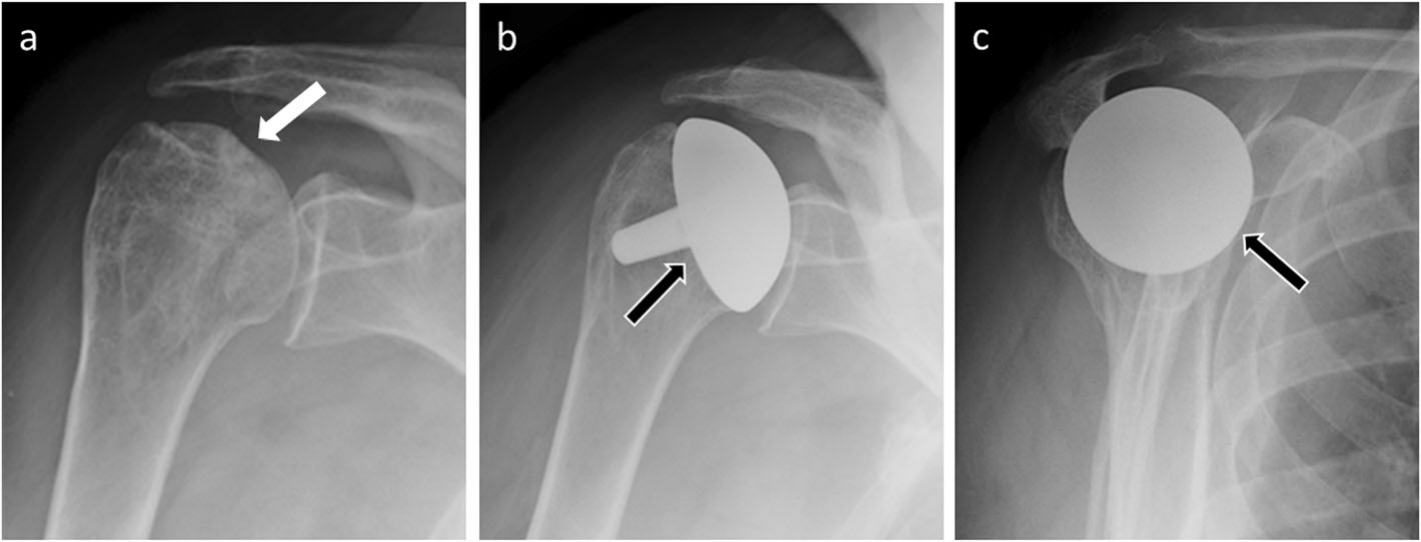
**a** Anteroposterior radiograph in a 63-year-old woman shows avascular necrosis of the humeral head (white arrow). Anteroposterior (**b**) and scapular Y (**c**) radiographs of normal findings in the same patient as in **a** with humeral head resurfacing arthroplasty. The humeral prosthetic head (black arrows) is centered within the native glenoid

On postoperative radiographs, the humeral prosthetic head should be centered within the native glenoid, just as the humeral prosthetic stem should be centered within the humeral shaft for hemiarthroplasties [8] (Fig. 5).

## Imaging tools for radiological shoulder prosthesis exploration

### Standard radiography

Due to its easy availability, low cost, and reproducibility, standard radiography is the core of shoulder arthroplasty monitoring imaging and first-line imaging modality. Two to five X-ray views are commonly used, depending on local surgical and radiological preferences: anteroposterior views in different rotations, a scapular Y view (Neer’s view), and an axillary view (cross-table view) [8, 9].

There is no consensus on the rhythm of systematic monitoring of shoulder prostheses in radiography in asymptomatic patients: usually, a first assessment is performed within the first 3 to 6 weeks, then a second between 3 months and 1 year, before continuing with an annual radiographic assessment [10].

When a complication is suspected in a symptomatic patient, radiography should be performed first and especially before other more advanced cross-sectional imaging modalities. During long-term follow-up, standard radiographs allow demonstration of prosthetic loosening, superior humeral migration (evidence of rotator cuff failure), acute or stress-related periprosthetic fractures, and advanced wear of the glenoid in case of partial joint replacement.

### Ultrasound

Sonography is increasingly used because it is easily available and not limited by hardware-related artifacts (unlike computed tomography and magnetic resonance imaging). Ultrasound is validated for the analysis of periarticular soft tissue disorders [11]. It also allows a dynamic evaluation of the shoulder. Rotator cuff tears, bicep tendon pathology (tear or tenosynovitis), and intra-articular and soft tissue infection (intra-articular effusion or periarticular collections) can be detected by ultrasound after shoulder arthroplasty. New ultrasound modalities have recently been developed to assess the deltoid muscle integrity after RSA: contrast-enhanced ultrasound (CEUS) for the study of perfusion and acoustic radiation force impulse (ARFI) for that of elasticity. The operated-on deltoid muscle has a higher stiffness than contralateral muscle and deltoid reduced perfusion appears to be associated with limited range of motion and below-average outcome [12].

### Computed tomography

In case of shoulder arthroplasty complication suspicion, when standard radiographs are not contributive or normal, computed tomography (CT) plays an important role in the diagnostic procedure. This second-line imaging modality allows analysis of prosthetic component positioning; periprosthetic bone and soft tissues, particularly for detecting periprosthetic radiolucent lines, collections, rotator cuff tears; or muscular trophicity anomalies. The use of scanners with technologies reducing hardware-related artifacts and irradiation should be favored, particularly for periprosthetic soft tissue analysis. From a technical point of view, it is necessary to increase tube voltage and current, and use soft tissue reconstruction algorithm [9].

More recently, dual-energy CT has been proposed to reduce beam-hardening artifacts by allowing the synthesis of virtual monochromatic spectral images [13]. But this technique does not avoid the effects of photon starvation—the X-ray beam becomes markedly attenuated after passing through metallic hardware, and insufficient numbers of photons reach the detectors—and its association with metal artifact reduction software seems more effective [14].

### Magnetic resonance imaging

Until recently, magnetic resonance imaging (MRI) has been considered uninterpretable in the exploration of articular metal prostheses, mainly because of artifacts generated by the high magnetic susceptibility of metallic prostheses. This susceptibility is responsible for proton spatial coding anomalies around the prosthesis. Nowadays, optimization of conventional sequences and development of dedicated sequences (VAT (view angle tilting), SEMAC (slice-encoding metal artifact correction), and MAVRIC (multiacquisition variable-resonance image combination)) make it possible to limit artifacts and encourage the use of MRI. In conventional MRI, the use of 1.5-T machines and spin echo sequences will be preferred, with small slice thickness, and high matrix and bandwidth. For fat suppression, a short tau inversion recovery (STIR) sequence should be performed, because it is less sensitive to local field inhomogeneities in the presence of metallic hardware than chemical shift selective fat suppression technique. It should be noted that there is no absolute contraindication to MRI for these patients with shoulder prostheses (even with older prostheses).

In daily practice, it is preferable to use T1-weighted sequences coupled with dedicated sequences (e.g., MAVRIC-T1) to obtain an “anatomical” view of the shoulder, and STIR sequences (also preferably coupled with dedicated sequences) to obtain an equivalent of T2-weighted sequences. MRI can detect several shoulder arthroplasty postoperative complications, such as infections, neuropathy, component loosening, tendon and muscle abnormalities, or glenoid wear in case of partial joint replacement [15]. Thus, intra-articular effusion, periarticular collections, tendon ruptures, denervation muscle edema, and periprosthetic lines on STIR sequences (hyperintensities) will be sought; on T1-weighted sequences, periprosthetic fractures and muscular fat involution (indirect sign of rotator cuff tear or deltoid dysfunction) will be identified.

## Complications common to all shoulder prostheses

### Infection

Periprosthestic joint infection (PJI) of the shoulder is a rare but serious complication with an incidence of 0.98% in the USA [16]. It is most frequently seen after reverse arthroplasty probably due to hematoma formation, the so-called dead space associated with the lack of a rotator cuff, advanced patient age, and prior shoulder surgery [17]. Risk factors associated with periprosthetic shoulder infections are endogenous (diabetes, obesity, immunosuppression, oncological diseases, rheumatoid arthritis, previous or chronic infections, and bacteriuria) and exogenous (extended duration of the operation, blood transfusion, and hypothermia) [18].

The main risk factors for infection after RSA are surgical history of the shoulder (e.g., rotator cuff surgery), history of a prior failed arthroplasty, and age younger than 65 years [19]. After TSA, the main risk factors are male gender and younger age [20]. After shoulder hemiarthroplasty, an underlying diagnosis of trauma is associated with a higher risk of PJI [21]. Microorganisms most commonly associated with PJI are the skin pathogens including *Staphylococcus aureus* and *Cutibacterium* (formerly *Propionibacterium*) *acnes*, probably because of the proximity of the surgical site to the axillary region [22]. Successful treatment is hampered because clinical findings may be subtle, many of the traditional signs of infection not being present, and cultures frequently not positive for as long as 2 weeks [23].

**Fig. 6:**
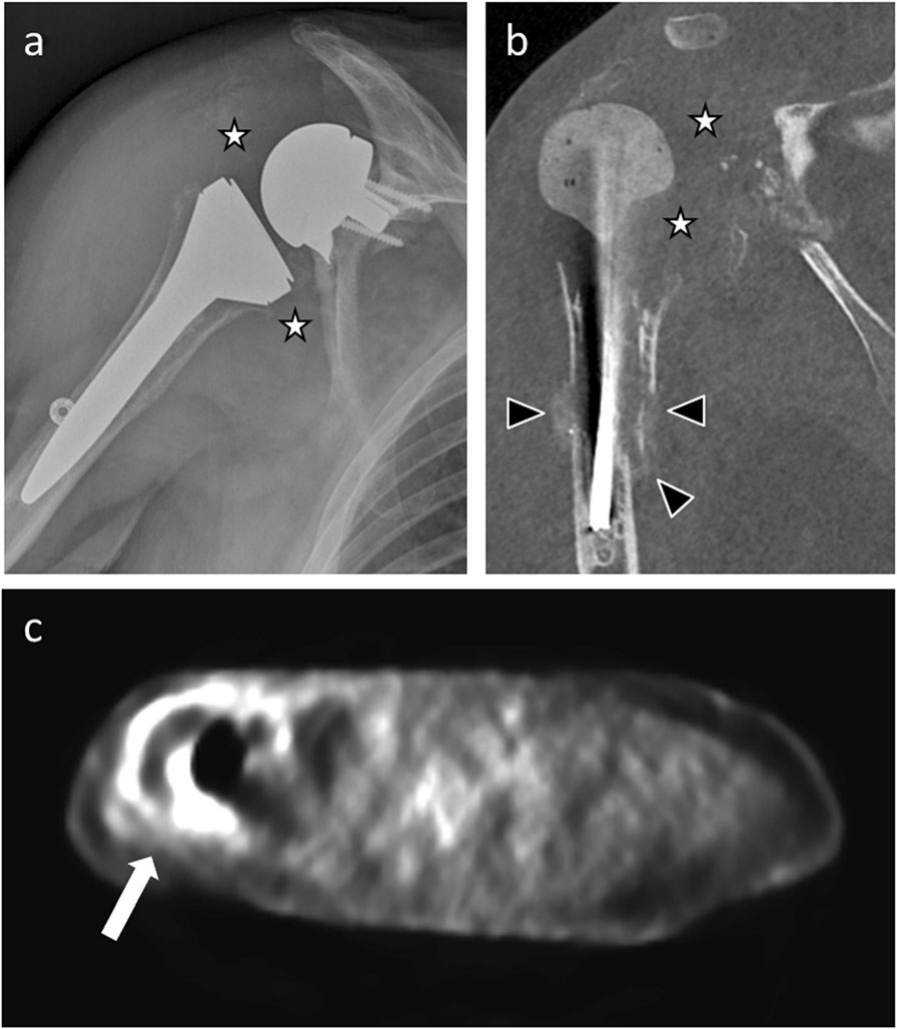
**a** Anteroposterior radiograph of a 73 years-old man with periprosthetic joint infection shows a soft-tissue thickening (white stars) around the reverse shoulder arthroplasty. **b** Coronal CT image of the same patient as in **a**, immediately after surgical revision and placement of a spacer, shows the same soft-tissue thickening (white stars), focal cortical losses, and periosteal appositions (arrowheads). **c** Axial FDG-PET image of the same patient as in **a** before surgery shows abnormal uptake around the prosthesis

On radiographs, infection can result in progressive irregular lucency around the prosthesis and periosteal appositions. Unfortunately, these signs appear at an advanced stage of the disease. Cross-sectional imaging techniques, such as CT and MRI, add often limited value in the presence of metallic prosthetic implants owing to beam-hardening and dephasing artifacts. Nuclear medicine studies may be used to investigate these patients. The two oldest radionuclide imaging modalities used for this purpose are bone scintigraphy and gallium citrate scintigraphy. 111In white blood cell (WBC) imaging in conjunction with 99mTc sulfur colloid marrow imaging should be sufficient [24]. Published results on the role of 18F-fluorodeoxyglucose-positron emission tomography (FDG-PET) for diagnosing PJI are inconclusive due to various factors. However, all these nuclear medicine techniques are sensitive but lack specificity, for example, increased “abnormal” uptake can be seen at the site of arthroplasty, related to bone remodeling, for up to 1 year after surgery [25]. A multi-modality approach including joint aspiration (which may be performed under fluoroscopic, ultrasound, or CT scan control), cross-sectional imaging (CT and MRI), and nuclear medicine studies is typically necessary to diagnose PJI (Fig. 6).

Two-stage reimplantation for prosthetic joint infection reportedly has the lowest risk for recurrent infection [26].

### Stress shielding and periprosthetic fractures

Periprosthetic humeral and glenoid fractures have a prevalence of 1.0%. They are observed in all types of shoulder arthroplasty but are more common after RSA. Periprosthetic fractures can be both intraoperative and postoperative, but the former are twice as frequent. Excessive reaming of the glenoid surface and screw penetration of the glenoid vault are incriminated for intraoperative glenoid fractures during RSA [27].

**Fig. 7:**
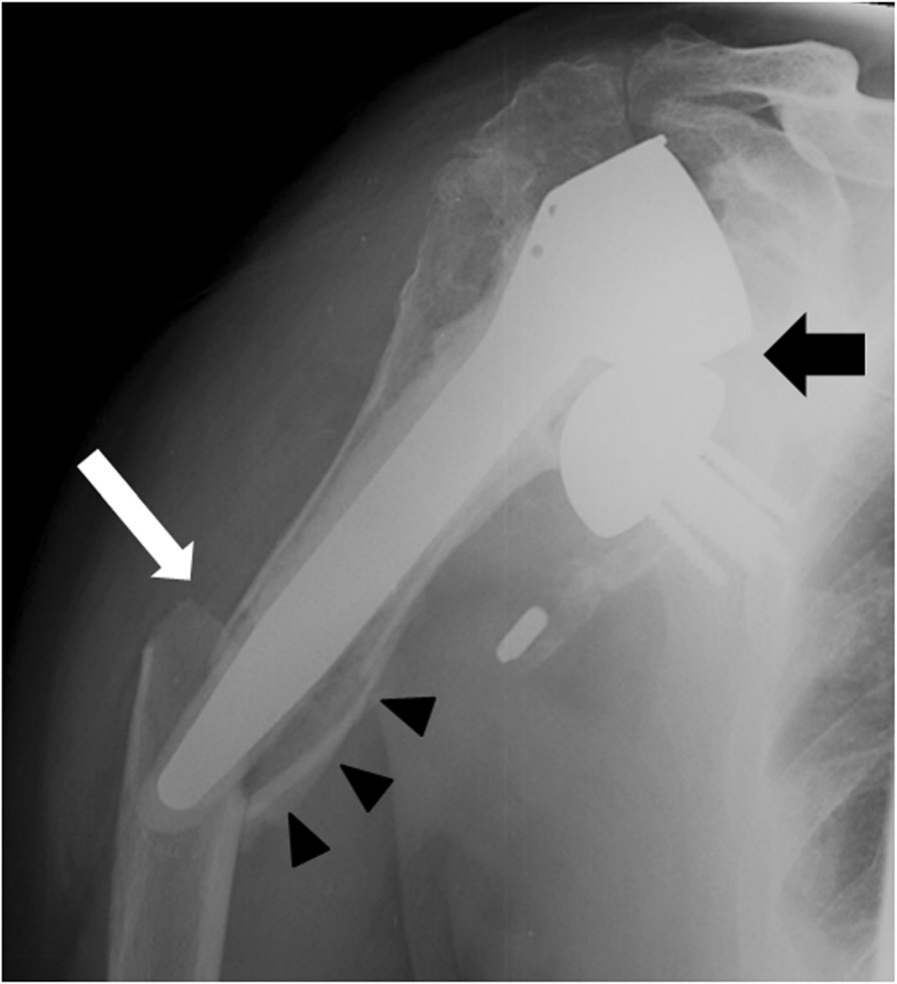
Anteroposterior radiograph of a 73-year-old man shows a periprosthetic fracture (white arrow) and a complex glenohumeral dislocation (black arrow). Note that there was previously a stress shielding manifested by cortical thinning and radiolucency area around the distal humeral stem (arrowheads)

Stress shielding is a predisposing risk factor for postoperative periprosthetic humeral fractures and is observed in 9% of the cases. It is characterized by bone adaptation to the modification of stress distribution, which causes bone thinning (external remodeling) or becoming excessively porous (internal remodeling). Osteoporosis could be a risk factor for stress shielding [28], as well as the relative stem size. Indeed, proximal stress shielding decreases with the use of short stems in total shoulder arthroplasty [29]. Radiographically, stress shielding is manifested by cortical thinning and increased central areas of radiolucency, reportedly more frequently in the proximal lateral humerus (Fig. 7).

Different classification systems for periprosthetic fractures are used, the most employed having been described by Wright and Cofield [30], who separate fractures into three types according to the position of the fracture in relation to the tip of the humeral component: type A, fracture at the tip of the stem extending proximally more than one third the length of the stem; type B, fracture at the tip but with less proximal extension; type C, fracture distal to the implant and fractures extending into the humeral metaphysis.

Treatment may be orthopedic or surgical depending on the implant stability and the patient’s general condition.

### Humeral component loosening

Humeral component failure is a rare complication of shoulder arthroplasty, with a prevalence of less than 1% [27]. Osteoporosis, rheumatoid arthritis, and rotator cuff tear arthropathy are classic risk factors. In addition, glenoid loosening and more precisely polyethylene wear seem to be associated with the development of humeral proximal bone remodeling and favor humeral component loosening [31].

**Fig. 8:**
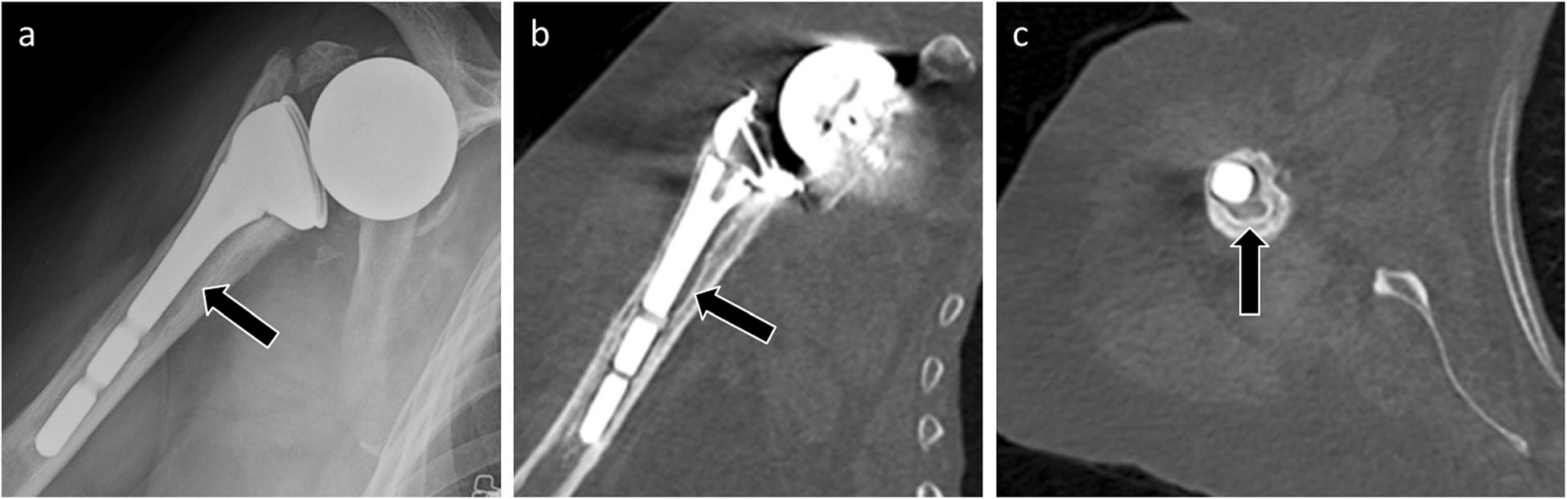
Anteroposterior radiograph (**a**) and coronal (**b**) and axial CT (**c**) images of a 79-year-old woman with humeral component loosening manifested by an irregular radiolucent line > 2 mm around the prosthetic stem (arrows)

Radiological diagnosis of humeral component loosening is based on research and analysis of periprosthetic humeral radiolucent lines. By analogy with the hip arthroplasties, Sperling described eight zones bordering a humeral implant. A humeral component is considered “at risk” when a radiolucent 2-mm-wide line or greater is present in three or more of the eight zones [32] (Fig. 8). Humeral implant tilt or subsidence sign loosening as well.

Recent studies showed that these radiological changes are less common with the use of stemless shoulder prosthesis [33] and that they do not affect the clinical mid-term outcome after stemless humeral head replacement [34].

### Heterotopic ossification

**Fig. 9:**
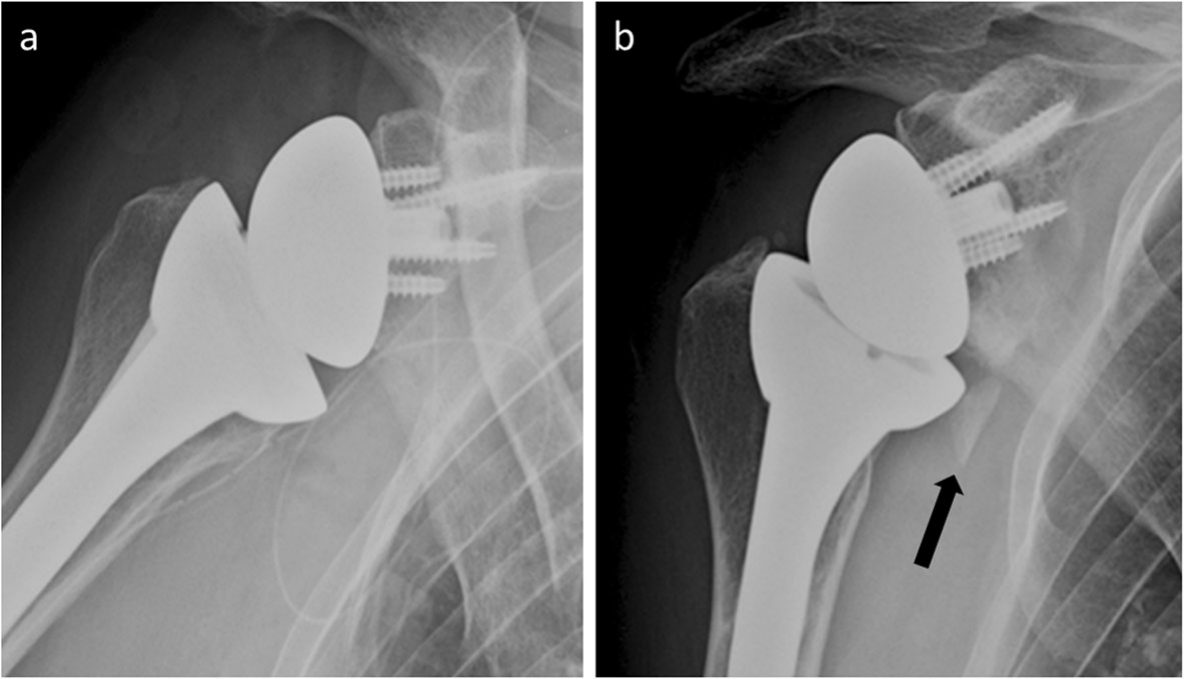
Anteroposterior shoulder radiographs of an 84-year-old woman with reverse shoulder arthroplasty. **a** Normal postoperative radiograph. **b** One year later, radiograph shows heterotopic ossification (arrow), with the patient asymptomatic

Heterotopic bone formation following shoulder arthroplasty is frequent and develops early after surgery (45%, 1 year after surgery) [35]. Heterotopic ossification (HO) is well analyzed in standard radiography (Fig. 9) and CT can provide more precise information on the anatomical location of bulky ossification that would be disabling.

Kjaersgaard established a visual scale for rating ossifications (grade 0 to grade 3) according to the space between the medial humeral shaft and the lateral glenoid [35]. HO is often low grade and does not significantly alter the functional prognosis [36]; in higher grades, a limited active elevation may be observed. Only patients with cuff tear arthropathy would have an increased risk of developing HO [37].

### Implant failure

**Fig. 10:**
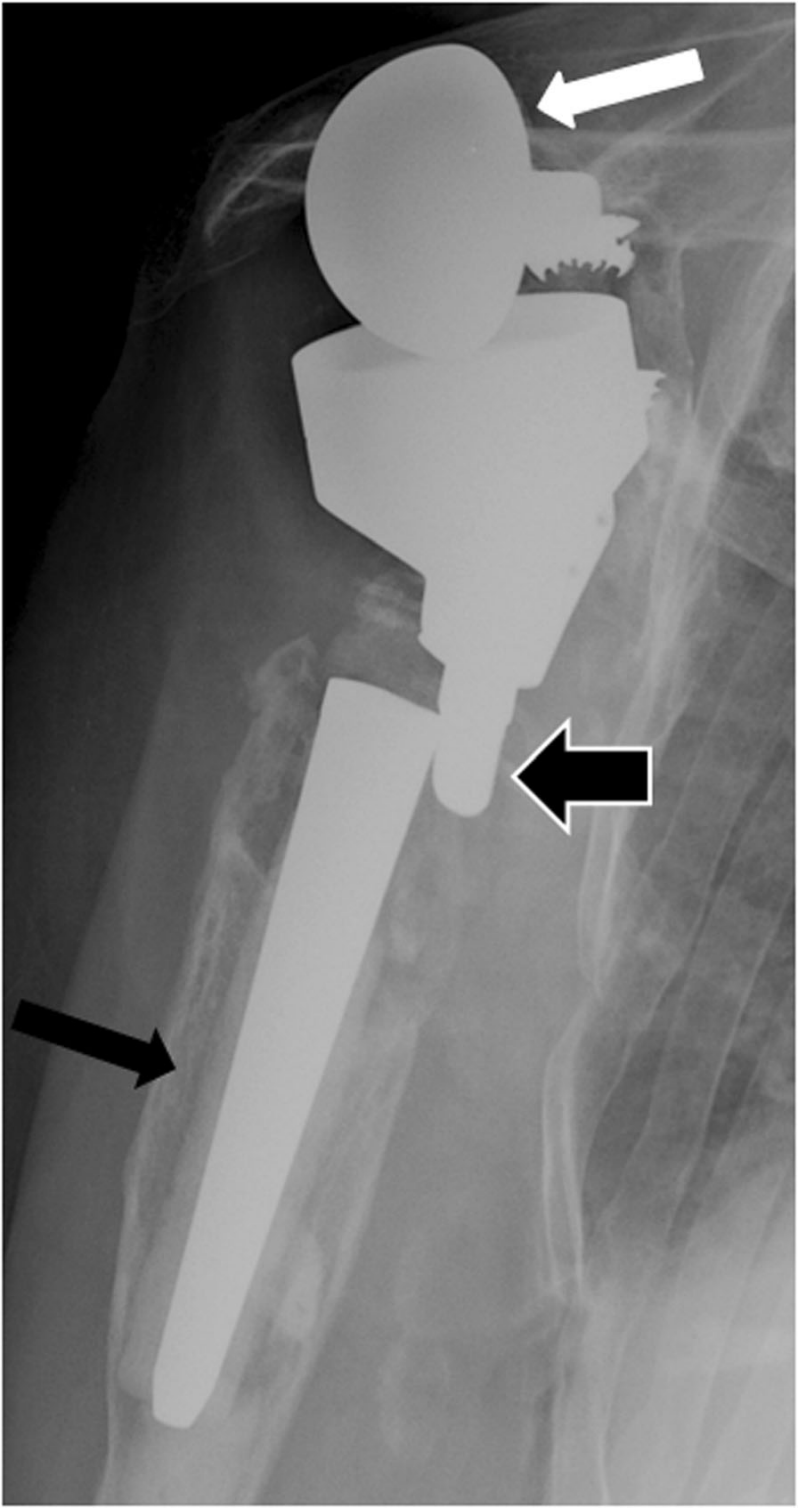
Anteroposterior radiograph of a reverse shoulder arthroplasty in a 73-year-old patient shows humeral implant disjunction with unscrewing of the two components (thick arrow). This is secondary to both humeral loosening resulting in chronic abnormal mobility of the implant (black thin arrow), and glenoid loosening with the glenoid component “wedged” under the acromion (white thin arrow)

**Fig. 11:**
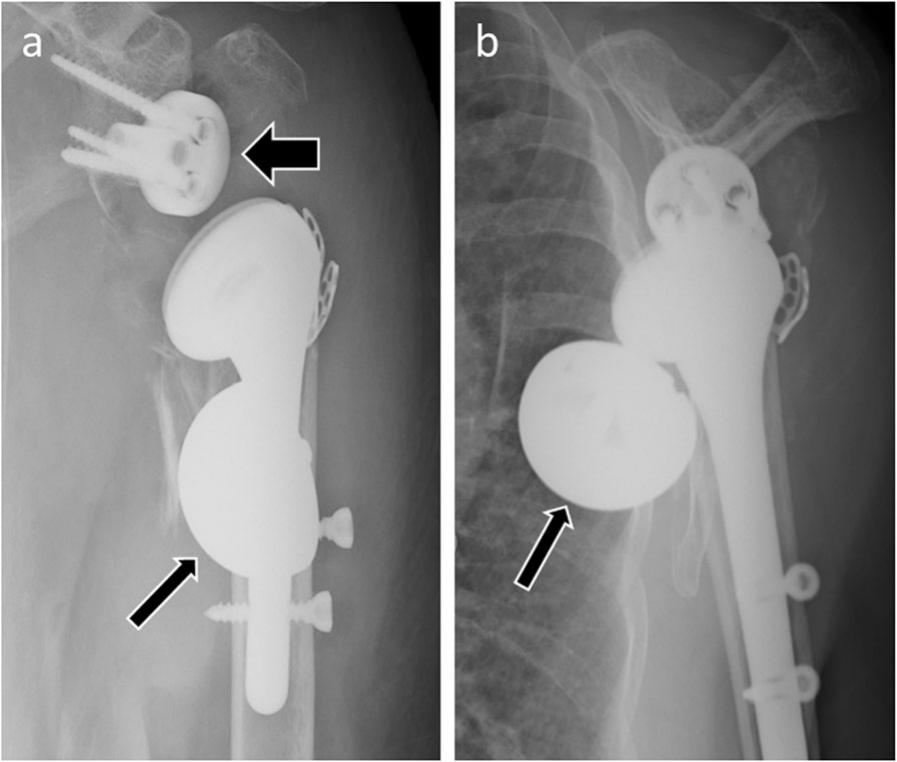
Anteroposterior (**a**) and scapular Y (**b**) radiographs of a 87-year-old woman, with reverse shoulder arthroplasty placed 4 days earlier, show glenosphere anteroinferior dislocation (thin arrows). Note the emptiness of the metaglene (thick arrow)

Implant failure is a rare complication, but usually imposes surgical revision. Subluxation or dislocation of polyethylene inlays, broken fixations screws, fracture of the keel or metal glenoid backing, dissociation of the polyethylene glenoid insert from its metal tray, dislocation of the humeral component (Fig. 10), and dissociation of the glenosphere from its metaglene (Fig. 11) can be observed. All these situations usually require surgical revision.

### Nerve injury

Nerve damage affects axillary nerve preferentially, more rarely the brachial plexus. It frequently results from nervous stretching during luxation-reduction maneuvers performed during surgery. Most axillary lesions are fortunately rare and resolving [38]. Deltoid muscle dysfunction is a serious complication secondary to either axillary nerve involvement or deltoidal disinsertion (after the upper lanes). It commonly leads to reduced abduction and inferior instability. Ultrasound and MRI may be used for the diagnosis of deltoid muscle abnormalities [11, 12, 15].

## Specific complications of different shoulder prostheses

### Reverse shoulder arthroplasty

#### Instability following reverse shoulder arthroplasty

Instability is the most common complication after RSA and accounts for 31.3% of all complications of these arthroplasties [27]. Instability occurs in an anterior-superior direction (opposite of anterior-inferior glenohumeral dislocation on the native shoulder) after a combination of adduction, extension, and internal rotation of the arm. Dislocation is secondary to unopposed deltoid contraction, and therefore, anything causing a suboptimal deltoid tensioning is a risk factor. Deltoid dysfunction from rupture or incorrect surgical technique, acromial fracture, and axillary nerve injury are common causes [27].

**Fig. 12:**
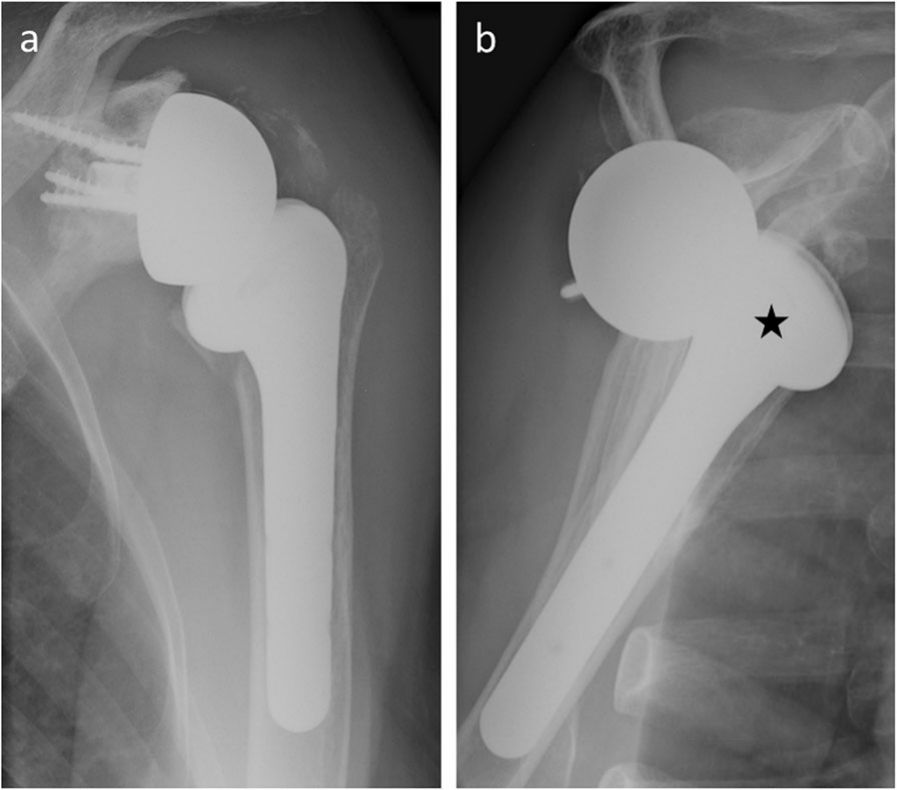
Anteroposterior (**a**) and scapular Y (**b**) radiographs of a 76-year-old woman with reverse shoulder arthroplasty (2 months after surgery) show an evident anterior dislocation (star) in scapular Y view. Note that this dislocation is difficult to highlight on anteroposterior view

Diagnosis is usually easy on standard radiographs, with the help of the anteroposterior, scapular Y, and axillary views (Fig. 12). The humeral component dislocates anteriorly (on scapular Y and axillary radiographs) and superiorly (on anteroposterior radiographs), along the direction of the deltoid muscle [7].

#### Scapular notching

Scapular notching is a specific complication to RSA, with a reported incidence of 50 to 96% [39]. From a biomechanical point of view, scapular notching would reflect a mechanical impingement of the humeral cup on the lateral scapular pillar during adduction, related to medialization of the shoulder’s rotation center. However, the clinical implications of notching and its relationship with glenoid loosening are controversial, with discordant results according to studies, although scapular notching seems to worsen over time [40, 41].

**Fig. 13:**
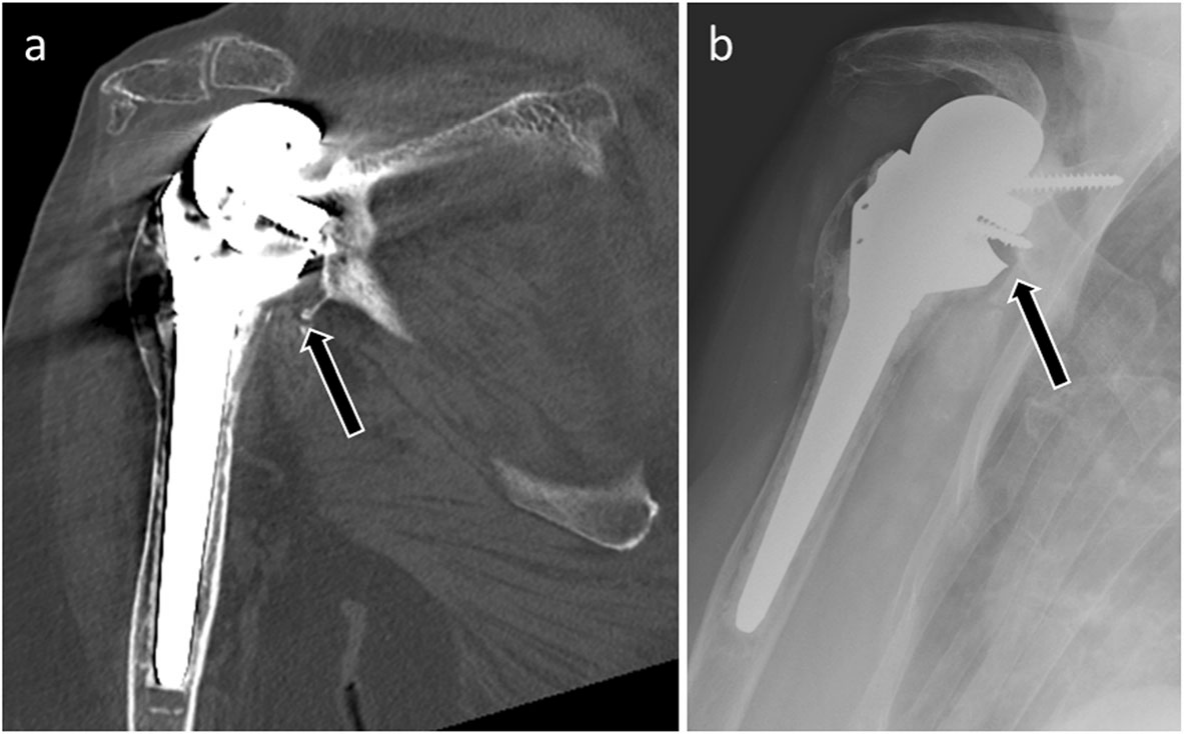
Coronal CT image (**a**) and anteroposterior radiograph (**b**) of an 89-year-old patient with painful reverse shoulder arthroplasty show scapular notching manifested by bone resorption of the inferior scapular border (arrows)

On radiographs and CT, we observe bone resorption of the inferior scapular border (Fig. 13). The extent of the notch was classified in relation to the inferior screw of the glenosphere, from grade 0 to 3 (Sirveaux classification): 0, absence of notch; 1, small notch stopping short of inferior screw; 2, medium notch reaching inferior screw; 3, large notch extending beyond inferior screw [42]. Inferior positioning of the glenosphere, inferior tilting of the glenosphere, and a lateralized center of rotation are surgical strategies to reduce scapular notching rate [40]. These elements are therefore to be well analyzed on standard radiographs.

#### Scapular spine and acromion fractures

Scapular spine and acromion fractures are specific complications to reverse total shoulder arthroplasty, with a prevalence of 1% [27]. They are more commonly seen in osteopenic patients (osteoporosis) [43]. These fractures seem to be responsible for lower mid- and long-term clinical outcomes and increase the risk of prosthetic revision [44].

**Fig. 14:**
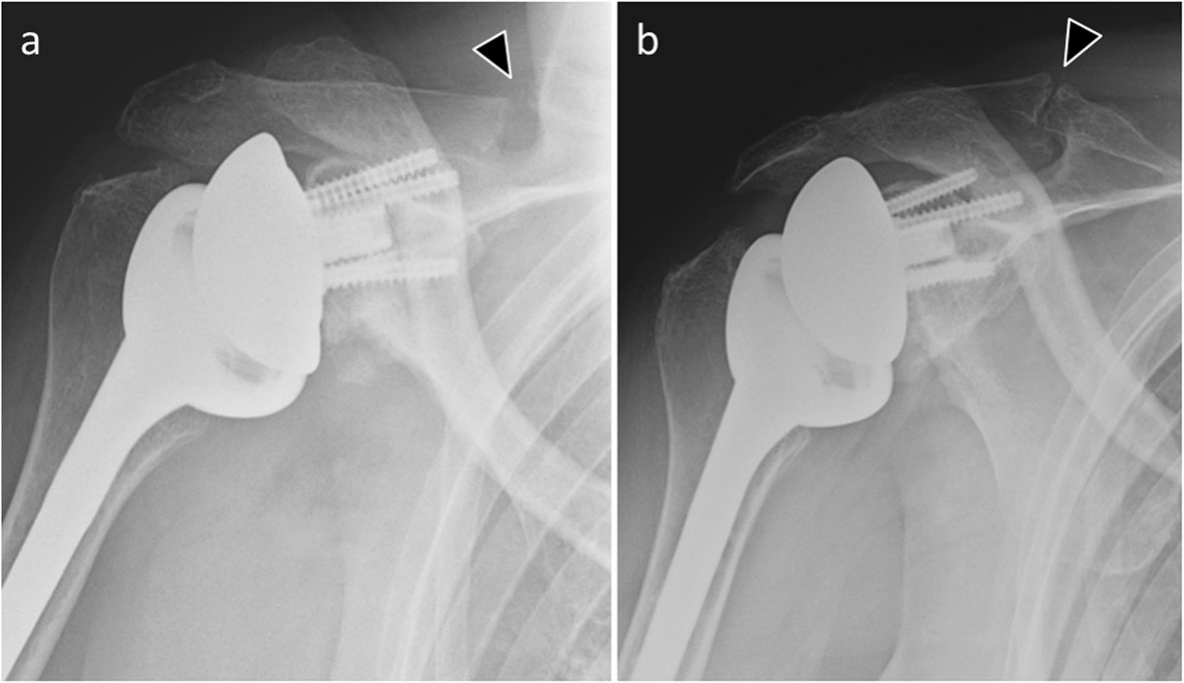
Anteroposterior radiographs of a 67-year-old woman with reverse shoulder arthroplasty. **a** Radiograph shows a proximal scapular spine fracture (arrowhead). **b** This fracture is more visible after a few weeks due to pseudoarthrosis (arrowhead)

A radiological classification has been proposed, ranging from types I to III, depending on the location of the fracture line regarding the origin of the deltoid muscle [45]: type I, involvement of a portion of the anterior and middle deltoid origin; type II, at least the entire middle deltoid origin with a portion but not all of the posterior deltoid origin; type III, the entire middle and posterior deltoid origin. The diagnosis is sometimes difficult in standard radiography, and CT is often used to detect these fractures (Fig. 14). Decreasing acromion-to-tuberosity distance and increasing acromial tilt on consecutive radiographs may improve fracture detection [43].

### Total shoulder arthroplasty

#### Glenoid component loosening

**Fig. 15:**
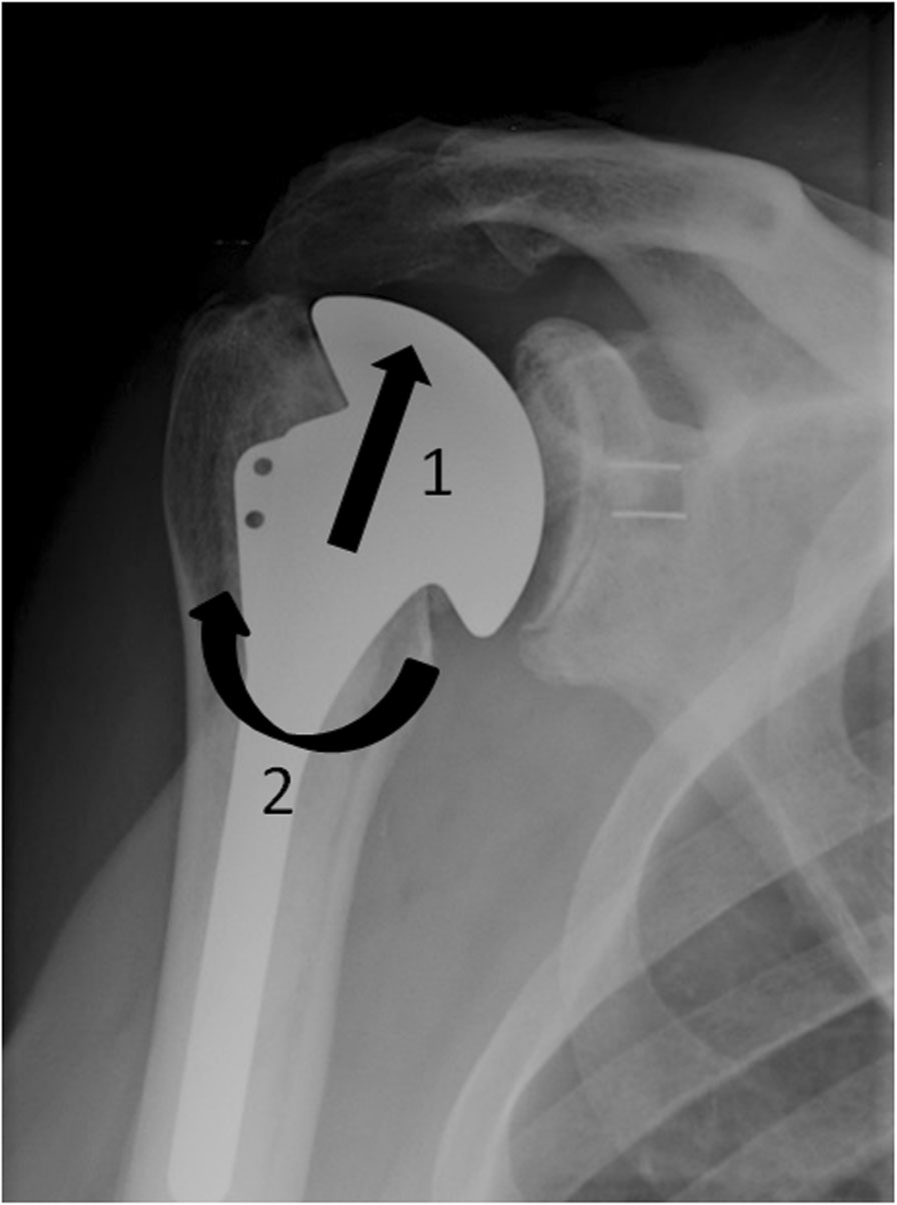
Anteroposterior shoulder radiograph in a patient with total shoulder prosthesis shows the “rocking horse” effect: a superior humeral migration (arrow 1) associated with repetitive abduction movement (arrow 2) may generate superior tipping of the glenoid component favoring glenoid loosening

Glenoid component loosening is the most common complication of total shoulder arthroplasty (about 38% of complications after TSA) [27]. Its origin is multifactorial. Rotator cuff deficiency is responsible for a superior humeral component displacement. This superior migration generates superior tipping of the glenoid component, called “rocking horse” glenoid [46] (Fig. 15). Glenoid implantation in a position incompatible with the anatomical version of the glenoid, as during prosthetic implantation on osteoarthritic joint with advanced erosion of the posterior glenoid, is also the source of excessive eccentric forces favoring glenoid loosening.

**Fig. 16:**
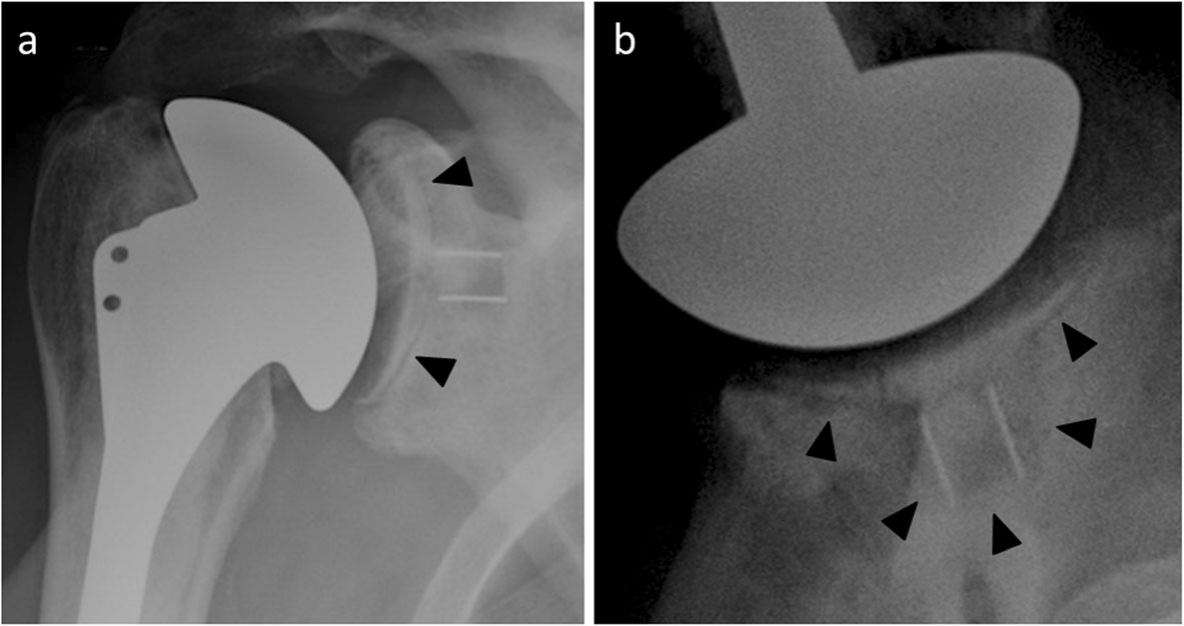
Anteroposterior (**a**) and axillary (**b**) radiographs in a 66-year-old man with shoulder pain show a complete radiolucent line around the glenoid component revealing glenoid loosening

Radiographically, glenoid loosening is defined as glenoid component migration, tilt, or shift or as a complete radiolucent line more than 1.5 mm thick [17] (Fig. 16). Predictive radiological scores of loosening have been described; the most commonly used is the Molé score [47]. This score (maximum at 18) is applied to the analysis of 6 zones around the glenoid component, with allocation of 1 to 3 points depending on the radiolucent line thickness: a score above 12 indicates probable loosening, between 6 and 11 possible, and below 6 as impossible.

**Fig. 17:**
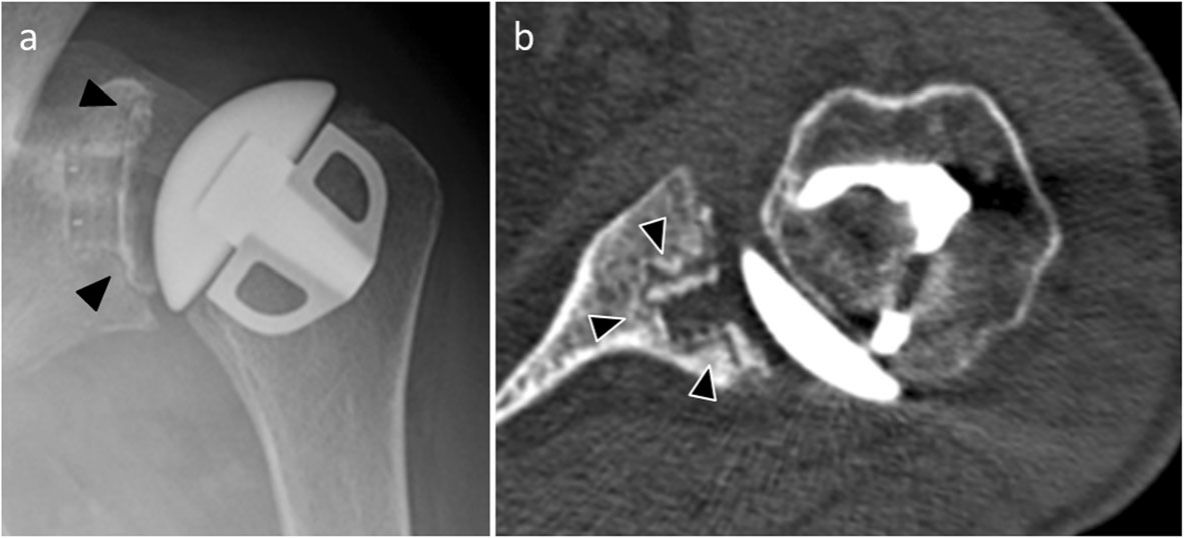
**a** Anteroposterior shoulder radiograph of a 61-year-old man with total shoulder arthroplasty shows a radiolucent line around the glenoid component (black arrowheads). **b** Axial CT image in the same patient as **a** allows a more precise analysis of this radiolucent line (black and white arrowheads).

CT is more sensitive than conventional radiography to detect and analyze the radiolucencies around the glenoid component (Fig. 17). This imaging modality is also more reproducible [48]. Gregory described a specific patient position to reduce the hardening artifacts that may interfere with the glenoid loosening assessment: patient in lateral decubitus to three-quarter decubitus position, allowing the scapula to tilt and the shoulder to be in maximal forward flexion [49].

**Fig. 18:**
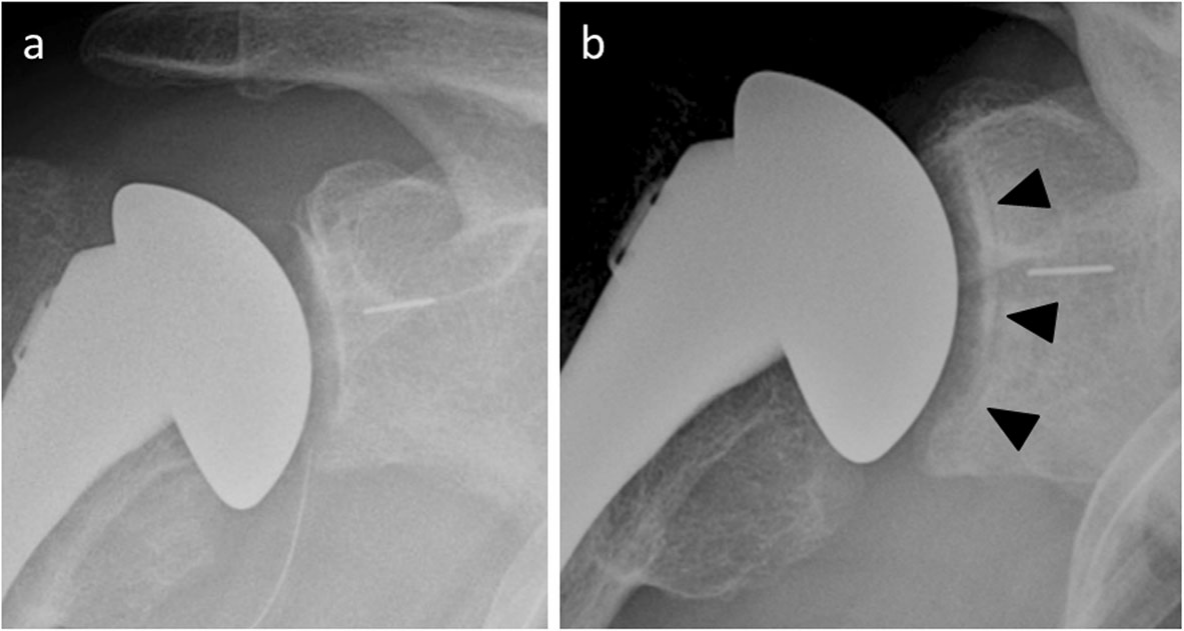
**a** Normal postoperative anteroposterior shoulder radiograph of a 51-year-old man with total shoulder arthroplasty. **b** Anteroposterior radiograph in the same patient as **a** 4 years later shows a radiolucent line around the glenoid implant (arrowheads), while the patient is asymptomatic

The correlation between glenoid component loosening and radiolucencies is discussed. Indeed, incomplete periprosthestic radiolucent lines or radiolucent lines less than 1.5 mm thick are commonly seen (Fig. 18). Asymptomatic radiolucent lines occur at a rate of 7.3% per year after primary shoulder replacement [50]. In addition, while keeled glenoid components have greater rates of asymptomatic radiolucent lines compared with pegged glenoid components [50], pegged components are associated with a lower revision risk compared with keeled components [51]. This inverse relationship is similar between all-polyethylene components and metal-backed components [52]. Therefore, the interpretation of such radiological abnormalities must be confronted with clinical data and the evolution of patients. In daily practice, a radiological diagnosis of glenoid component loosening implies close clinical and radiological monitoring, with prosthetic revision only considered if the patient is symptomatic and functionally limited.

#### Rotator cuff tear

Rotator cuff tears account for 9.0% of all complications after TSA [27]. These are serious complications because the shoulder prostheses cannot perform their biomechanical function without rotator cuff integrity. Multiple risk factors are known: insufficient tendon fixation after arthroplasty, oversized prosthesis, malrotation of the humeral component, multiple surgery, aggressive physiotherapy involving external rotation during the early postoperative period, and tendon compromise in humeral lengthening [9].

Subscapularis insufficiency is the most common rotator cuff abnormality after anatomic total shoulder arthroplasty and it is responsible for anterior instability [53]. The quality of the detachment and repair of the subscapularis tendon during prosthetic surgery is a key factor to avoid inadequate tendon healing.

**Fig. 19:**
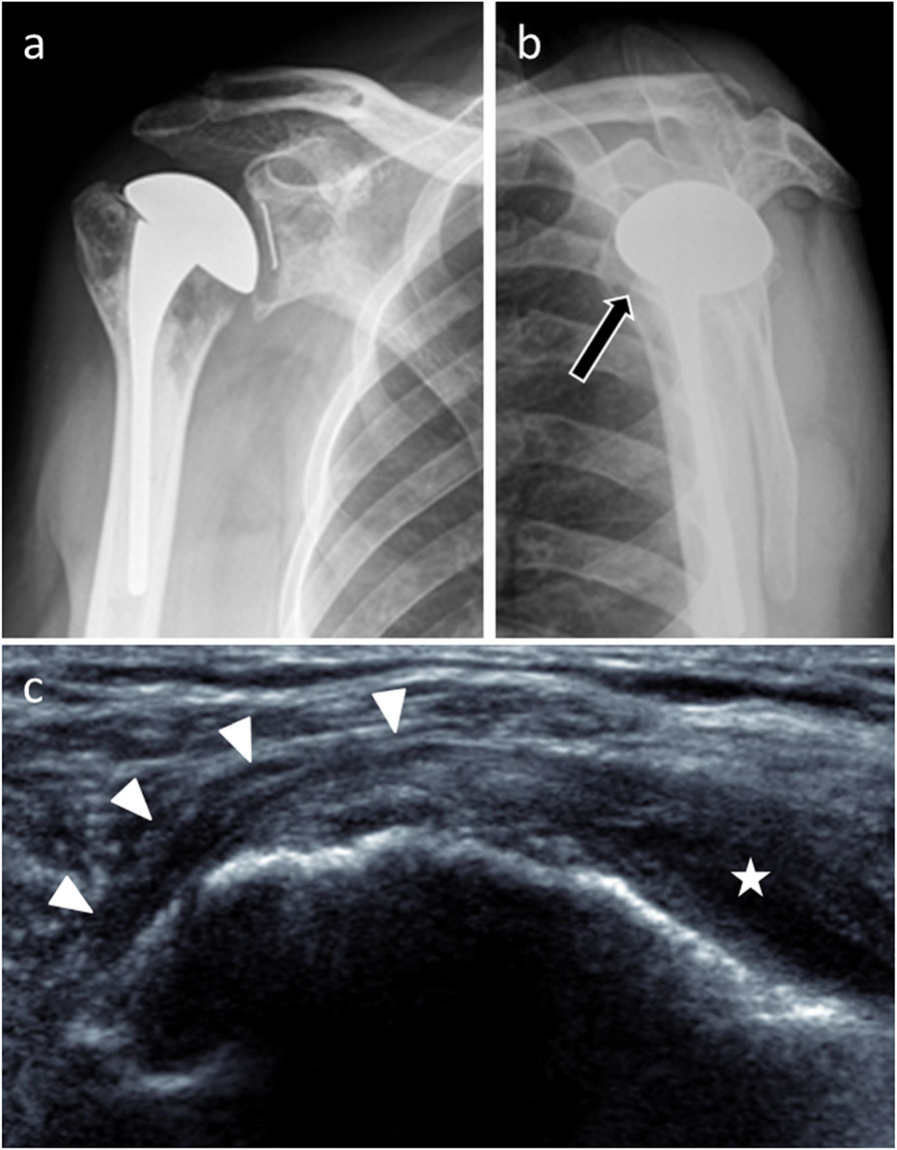
Anteroposterior (**a**) and scapular Y (**b**) radiographs of a 53-year-old man with total shoulder arthroplasty shows a slight anterior translation of the humeral head (arrow). Transverse grayscale US image (**c**) of the same patient shows subscapularis tendon rupture (arrowheads) with a retracted tendinous stump (star)

**Fig. 20:**
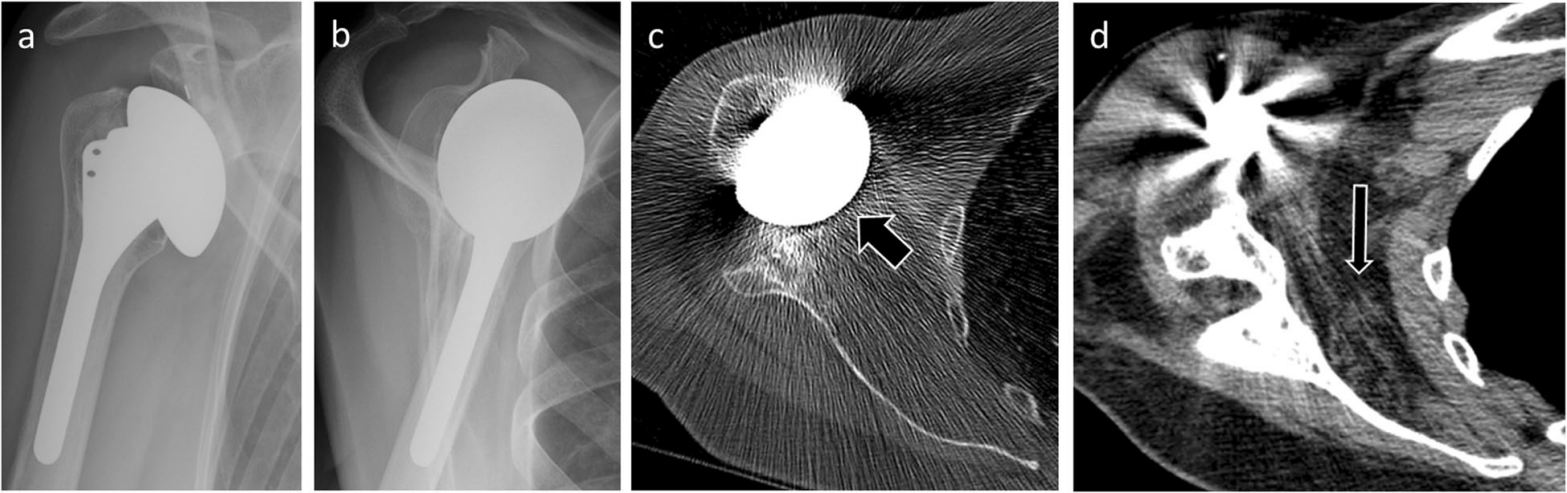
Anteroposterior (**a**) and scapular Y (**b**) radiographs in a 52-year-old woman with total shoulder arthroplasty and anterior instability show an anterior translation of the humeral head. **c** Axial CT image confirms this anterior translation (thick arrow). **d** Axial CT image with smooth reconstruction filter shows fatty degeneration of the subscapularis muscle (thin arrow). All these elements lead to suspicion of subscapularis tendon tear and this tear was confirmed during surgery

In imaging, ultrasonography helps diagnosis when it directly visualizes the tear [11] (Fig. 19). MRI also contributes to this as long as susceptibility artifacts do not interfere with interpretation. In CT, rupture usually cannot be seen directly because of the hardware artifacts, but a fatty degeneration of the rotator cuff is considered as a reliable indirect sign of a rotator cuff tear (Fig. 20). On standard radiographs, supraspinatus tear is suspected when the distance between the top of the humeral prosthesis and the acromion is less than 5 mm [9]. Anterior translation of the humeral head on anteroposterior and scapular Y views will suggest a subscapularis insufficiency.

### Humeral hemiarthroplasty and humeral head resurfacing arthroplasty

#### Progressive wear of the glenoid

**Fig. 21:**
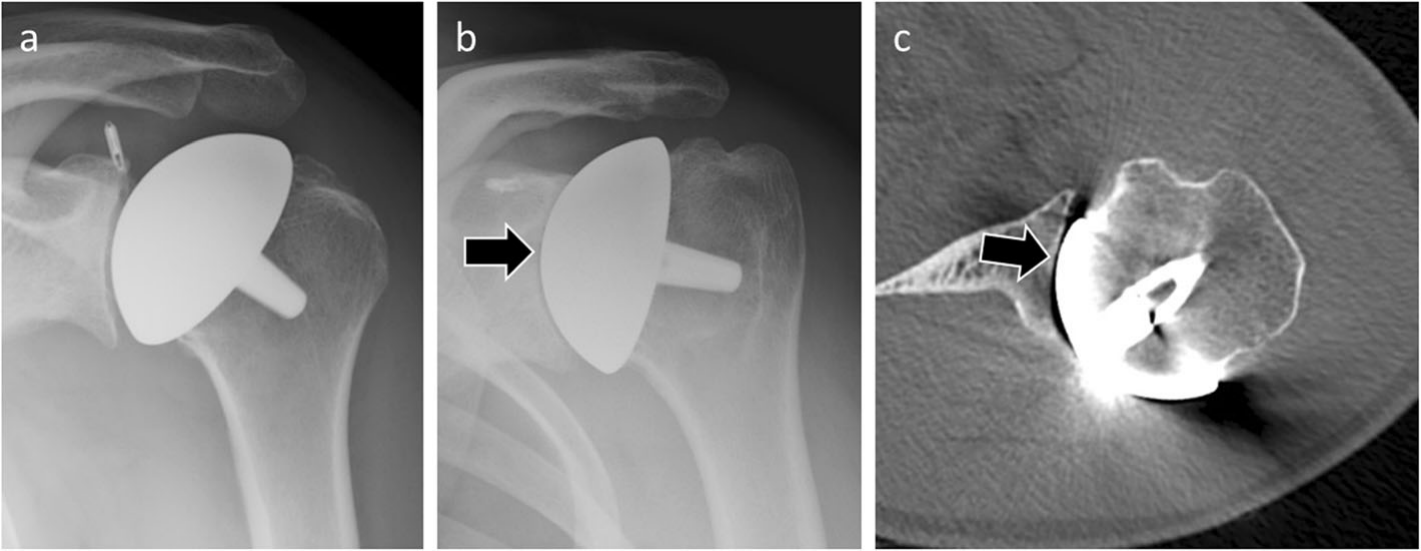
**a** Anteroposterior radiograph of a 36-year-old man with humeral head resurfacing arthroplasty. Anteroposterior radiograph (**b**) and axial CT image (**c**) in the same patient as **a**, 7 years later, show native glenoid wear manifested by narrowing of the glenohumeral spacing (arrows)

The unique specific complication of the partial shoulder joint replacement is the progressive wear of the native glenoid. Indeed, replacement of the humeral head modifies biomechanical constraints on the glenoid, leading to the long-term development of osteoarthritic remodeling. In this case, prosthetic revision will then be proposed, with, generally, conversion to total shoulder arthroplasty. The radiological diagnosis will be made on the successive standard radiographs, showing a progressive narrowing of the glenohumeral spacing over a few months, associated with posterior glenoid wear [54] (Fig. 21).

## Conclusion

Currently, shoulder arthroplasties are orthopedic implants commonly used in orthopedic surgery. These prostheses can be total shoulder arthroplasties, reverse shoulder arthroplasties, or partial shoulder joint replacements. Each of them may have specific complications, and the radiologist must be able to detect them to initiate quick and appropriate management (Table 1). Standard radiographs are the basis of shoulder arthroplasty imaging and are performed before cross-sectional imaging examinations. CT, MRI, ultrasound, and nuclear medicine techniques (scintigraphy, FDG-PET) are actually used if the radiographs are normal or inconclusive in case of suspicion of complication.

**Table 1:** Summary table of the different complications of shoulder arthroplasties

All types of shoulder prosthesis	Reverse shoulder arthroplasty	Total shoulder arthroplasty	Partial shoulder joint replacement
Infection	Instability	Glenoid component loosening	Progressive wear of the native glenoid (osteoarthritis)
Stress shielding and periprosthetic fractures			
Humeral component loosening	Scapular notching		
Heterotopic ossifications		Rotator cuff tear	
Implant failure	Scapular spine and acromion fractures		
Nerve injury			

This summary table presents the complications common to all shoulder arthroplasties (first column) and those specific to reverse shoulder arthroplasty (second column), total shoulder arthroplasty (third column), and partial shoulder joint replacement (fourth column)

## Data Availability

Not applicable.

## Abbreviations

ARFI: Acoustic radiation force impulse
CEUS: Contrast-enhanced ultrasound
CT: Computed tomography
FDG-PET: Fluorodeoxyglucose-positron emission tomography
HO: Heterotopic ossification
MAVRIC: Multiacquisition variable-resonance image combination
MRI: Magnetic resonance imaging
PJI: Periprosthetic joint infection
RSA: Reverse shoulder arthroplasty
SEMAC: Slice-encoding metal artifact correction
STIR: Short tau inversion recovery
TSA: Total shoulder arthroplasty
VAT: View angle tilting
WBC: White blood cell
